# Effect of neoadjuvant chemotherapy on intraoperative core temperature in patients with breast cancer: a retrospective cohort study

**DOI:** 10.1016/j.bjao.2022.100119

**Published:** 2022-12-31

**Authors:** Daniel D. Kim, Sarah M. DeSnyder, Patrick M. Dougherty, Juan P. Cata

**Affiliations:** 1 Department of Anesthesiology and Perioperative Medicine, The University of Texas MD Anderson Cancer Center, Houston, TX, USA; 2 Anesthesiology and Surgical Oncology Research Group, Houston, TX, USA; 3 Department of Breast Surgical Oncology, The University of Texas MD Anderson Cancer Center, Houston, TX, USA; 4 Department of Pain Medicine, The University of Texas MD Anderson Cancer Center, Houston, TX, USA

**Keywords:** breast cancer, intraoperative core temperature, mastectomy, neoadjuvant chemotherapy, neuropathy, vasopressors

## Abstract

**Background:**

Clinical evidence suggests that chemotherapeutic agents are associated with neuropathy and peripheral autonomic dysfunction. However, the possible effects of neoadjuvant chemotherapy on intraoperative temperature remain poorly characterised.

**Methods:**

We evaluated patients who underwent a mastectomy for breast cancer between April 2016 and July 2020. Propensity scores were used to match patients who received neoadjuvant chemotherapy with those who did not, and intraoperative core temperature patterns were analysed in the matched cohort. The independent associations between vasopressor use and heart rate during general anaesthesia in the matched cohort were also analysed.

**Results:**

Data from 1764 patients were analysed (882 patients in each group). Both groups presented a similar pattern of heat redistribution and subsequent rewarming; however, the neoadjuvant chemotherapy group did not reach the same intraoperative plateau temperature as the group that did not receive prior chemotherapy, with differences of up to 0.4°C (95% confidence interval: 0.11–0.63°C; *P*=0.005). In a subgroup analysis, neuropathy in patients who received neoadjuvant chemotherapy was associated with increased use of vasopressors and higher heart rate.

**Conclusions:**

In patients with breast cancer, neoadjuvant chemotherapy is associated with lower plateau core temperatures, increased vasopressor use, and higher heart rates during general anaesthesia, which is more severe in the presence of neuropathy.

Breast cancer is one of the most common types of cancer worldwide, with more than 2 million cases reported in 2018.^1^ Also, because of its increasing incidence in younger adults, breast cancer became the leading cause of cancer-related disability and years of life lost in women worldwide.^2^ Fortunately, the mortality rate has decreased over time with increased screening and advances in chemotherapy.^3^ Accordingly, neoadjuvant chemotherapy enables less aggressive treatments, such as breast-conserving surgery.^4^, ^5^

Unfortunately, chemotherapy is associated with several adverse effects. Of particular interest is chemotherapy-induced peripheral neuropathy.^6^ An experimental model showed that neurones of the autonomic nervous system are affected by chemotherapy agents.^7^, ^8^, ^9^ Dysautonomia can increase the risk of perioperative complications, such as delayed gastrointestinal emptying, altered cardiovascular response with diminished sympathetic nervous system activation, and intraoperative hypothermia resulting from decreased vasoconstriction.^10^

Although autonomic disturbances have been characterised in patients receiving adjuvant chemotherapy, little is known about the intraoperative consequences of neoadjuvant chemotherapy.^11^ Previous studies have demonstrated an association between intraoperative hypothermia and worse clinical outcomes, such as increased intraoperative blood loss,^12^ a higher rate of surgical wound infection, and a longer length of hospitalisation.^13^ However, whether neoadjuvant chemotherapy is associated with intraoperative hypothermia, thereby increasing the risk for perioperative complications, remains unknown.

We thus tested the primary hypothesis that neoadjuvant chemotherapy is associated with a lower core body temperature. Secondarily, we tested the hypothesis that neoadjuvant chemotherapy is associated with increased vasopressor use and heart rate during anaesthesia for mastectomy.

## Methods

### Study design and population

This was a retrospective cohort study of adult patients who underwent mastectomy for breast cancer at our institution between April 2016 and July 2020. The study was approved by the MD Anderson Cancer Center Institutional Review Board (IRB #2020–1261), and requirement for written consent was waived because this was a retrospective database study.

### Inclusion/exclusion criteria

We included patients who had their core body temperature measured during surgery. Patients diagnosed with diabetes mellitus before surgery were excluded because the disease can also cause dysautonomia and is independently associated with hypothermia during surgery, which would have confounded the results.^14^ Patients undergoing reoperation because of early recurrence, positive surgical margins, or emergency procedures were also excluded. Patients with duplicated or incomplete data (e.g. manual charting during electronic medical record system downtime) in the electronic medical record system were excluded. This article adheres to the Strengthening the Reporting of Observational Studies in Epidemiology (STROBE) guidelines (see the completed STROBE checklist in the Supplementary materials).

### Outcomes of interest

The intraoperative temperature was measured in the bladder, nasopharynx, or distal oesophagus using standard clinical thermometers. The temperature values were automatically captured every minute using an electronic medical record system. Virtually all patients had a forced-air warming blanket positioned across the lower extremities, which was activated after surgical draping without any pre-warming. Patients who received neoadjuvant chemotherapy underwent surgery within 4–6 weeks after their last chemotherapy cycle. Secondary outcomes included the use of any vasopressor (ephedrine or phenylephrine) during anaesthesia and the instantaneous heart rate measured by pulse oximetry or electrocardiography recorded every minute in the electronic medical record system. The neuropathy diagnosis was based on the International Statistical Classification of Diseases and Related Health Problems (ICD) codes registered in the patients' medical records. Patient anthropometric characteristics, intraoperative information, and hospitalisation data were obtained from the electronic medical record system.

### Statistical analysis

We compared the group that received neoadjuvant chemotherapy with the group that did not. We used descriptive statistics as appropriate (means and standard deviations, medians and quartiles, or proportions). Propensity score matching was conducted to ensure that any potential pre-surgical confounding variables were balanced between the groups. First, we predicted the probability of receiving neoadjuvant chemotherapy using a logistic regression model by including potential confounders as covariates. Patients who received neoadjuvant chemotherapy were matched with those who did not at a 1:1 ratio with a matching caliper within 0.15 standard deviations of the propensity score logit. The balance of each confounding variable was assessed based on the absolute standardised difference and was considered acceptable when it was lower than 0.15. The variables considered for the propensity score matching were age, sex, BMI, ethnicity, ASA physical status classification, duration of anaesthesia, and type of surgery (partial or radical mastectomy, unilateral or bilateral mastectomy, or breast reconstruction with flap).

Additionally, we compared other relevant perioperative variables not included in the propensity matching to reduce the chances of overfitting the logistic regression model. These included preoperative haemoglobin concentration, body surface area, estimated blood loss, and intraoperative fluid administered. Also, the Charlson Comorbidity Index and Elixhauser Comorbidity Index were calculated using the 10th revision of the ICD, coded within 1 yr of the patient's surgery date.^15^ Groups were compared using Student's *t*-test or the Wilcoxon–Mann–Whitney test, depending on data distribution. All tests were two-tailed, and the cut-off level for statistical significance was 0.05 for both the primary and secondary outcomes. SAS software version 9.4 (SAS Institute Inc. Cary, NC, USA) was used for all statistical analyses.

### Primary endpoint

To minimise the impact of artifacts on the temperature data,^16^ we excluded measurements below 32°C and above 38°C, any data whose collection was initiated more than 30 min after anaesthetic induction, and consecutive readings with temperature variations exceeding 0.5°C. After correcting for artifacts, the intraoperative temperature was summarised using spline regression and the distribution compared using Student's *t*-test or the Wilcoxon–Mann–Whitney test, depending on data distribution. Additionally, using the average temperature by time point, we determined the largest temperature difference between the groups.

A previous publication reported a difference of 0.1°C between the core temperatures of a large group of adult patients having noncardiac surgery and a subset of patients undergoing breast procedures using forced warming during surgery.^16^ Therefore, 0.1°C was established *a priori* as the minimum clinically important difference in our study.

### Secondary endpoint

The use of vasopressors between groups was analysed as a categorical and as a continuous variable. For categorical analysis, we compared the groups using the Cochran–Mantel–Haenszel test. Assuming that vasopressor use is a continuous variable with a log-normal distribution, we evaluated the difference between groups by comparing the ratio of geometric means using Student's *t*-test.

To reduce artifactual data from heart rate measurements, readings lower than 30 beats min^−1^ and higher than 160 beats min^−1^ were excluded. After correcting for artifacts, differences in heart rate between groups were summarised using spline regression and distribution, compared using Student's *t*-test or the Wilcoxon–Mann–Whitney test, depending on data distribution.

### Sample size and power calculation

To achieve 90% power to detect a difference of 0.1°C between the neoadjuvant and no chemotherapy groups, a sample of 1516 patients (758 in each group) was required.

## Results

In total, 3409 patients were included in the study (2527 in the no chemotherapy group and 882 in the neoadjuvant chemotherapy group). The baseline anthropometric, hospitalisation, and surgical data of all patients are summarised in Table 1. Before propensity score matching, patients who received neoadjuvant chemotherapy were younger, had more comorbidities, and experienced a longer duration of anaesthesia than those who did not receive neoadjuvant chemotherapy. Moreover, patients who received neoadjuvant chemotherapy had lower preoperative haemoglobin concentration (11.4 *vs* 13 g dl^−1^), a higher incidence of peripheral neuropathy (13.5% *vs* 5%), and more extensive operations (such as radical mastectomy and lymphadenectomy). No clinically relevant differences were found for intraoperative fluid administration, estimated blood loss, Charlson Comorbidity Index, or Elixhauser Comorbidity Index (Supplementary Tables 1, 3, and 4).

**Table 1:** Baseline anthropometric, hospitalisation, and surgical data before and after matching. Data are presented as mean [standard deviation] or *n* (%), as appropriate. ∗Propensity score of receiving neoadjuvant chemotherapy was estimated using a logistic regression model by including potential confounders as covariates. Patients were matched based on propensity scores at a 1:1 ratio. ^†^Variables with an absolute standardised difference >0.15 were considered imbalanced.

Variable	Before propensity matching			After propensity matching∗		
	No chemotherapy (*n*=2527)	Chemotherapy (*n*=882)	Absolute standardised difference^†^	No chemotherapy (*n*=882)	Chemotherapy (*n*=882)	Absolute standardised difference^†^
Age (yr)	61 [12]	56 [13]	0.37	56 [12]	56 [13]	0.01
Sex (female), *n* (%)	2512 (99)	880 (99)	0.06	877 (99)	880 (99)	0.05
Ethnicity (non-Hispanic), *n* (%)	2066 (82)	716 (81)	0.04	711 (81)	716 (81)	0.03
ASA physical status 2, *n* (%)	464 (18)	110 (12)	0.16	120 (14)	110 (12)	0.02
ASA physical status 3	2020 (80)	761 (86)		749 (85)	761 (86)	
Duration of anaesthesia (min)	273 [150]	296 [132]	0.16	306 [170]	296 [132]	0.08
BMI (kg m^−2^)	29 [7]	29 [7]	0.06	29 [7]	29 [7]	0.04
Partial mastectomy, *n* (%)	1379 (55)	362 (41)	0.21	405 (46)	362 (41)	0.05
Bilateral mastectomy, *n* (%)	163 (6)	45 (5)	0.04	73 (8)	45 (5)	0.14
Flap reconstruction, *n* (%)	233 (9)	91 (10)	0.04	112 (13)	91 (10)	0.08

After propensity score matching, 1764 patients were successfully matched (882 in each group). The absolute standardised difference was lower than 0.15 for all variables, showing that potential confounding variables were appropriately balanced between the groups (Table 1). Additionally, patients who received neoadjuvant chemotherapy presented a higher incidence of the uncomplicated hypertension component of the Elixhauser Comorbidity Index, although this was not clinically relevant (Supplementary Table 4).

**Fig 1:**
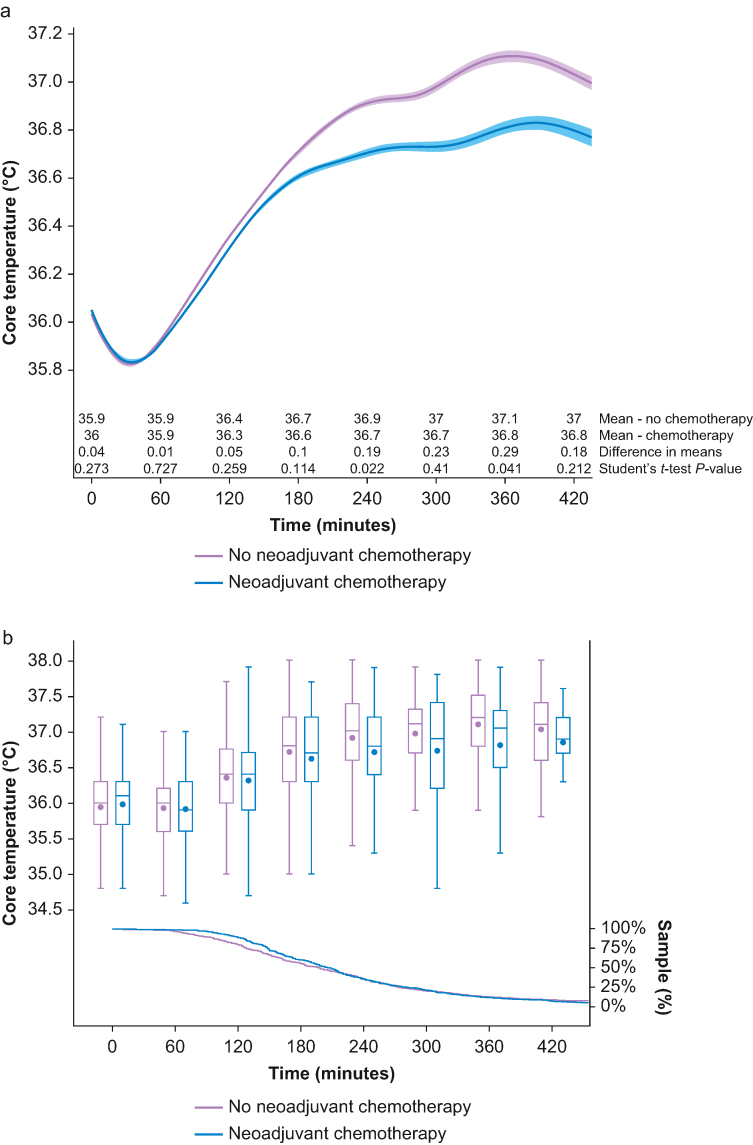
Comparison of core temperature between no neoadjuvant chemotherapy *vs* neoadjuvant chemotherapy groups using spline regression (represented by line) with 95% confidence interval (represented by band) as a function of time and hourly summary statistics (represented by box plot) with the proportion of sample data analysed over time (represented by line). (a) Comparison of core temperature over time between groups with hourly difference in means using Student's *t*-test. (b) Summary statistics (mean, median, inter-quartile interval, and minimum and maximum values) with proportion of sample data per group using Kaplan–Meier estimator.

In the matched cohort, both groups presented a similar pattern of decreasing temperature, reaching a nadir around 40 min after anaesthetic induction, a subsequent increase in core temperature for the remainder of the procedure, and a similar rewarming rate of 0.3°C h^−1^. Both groups reached a temperature plateau starting 3 h after the induction of general anaesthesia; thereafter, the neoadjuvant chemotherapy group presented lower temperatures than the no chemotherapy group (Fig. 1a). The Wilcoxon–Mann–Whitney test suggested that the temperature distribution differed between the groups (*Z*=–29.15; *P*<0.001). Close to 5 h after induction of anaesthesia, the proportion of validated temperature measurements decreased below 25% of the sample in each group (Fig. 1b).

A total of 1024 patients (58%) required at least one dose of vasopressor during surgery: 533 in the neoadjuvant chemotherapy group and 491 in the no chemotherapy group. There was an association between neoadjuvant chemotherapy and the use of vasopressors during surgery (odds ratio: 1.2; 95% confidence interval [CI]: 1.0–1.5; *P*=0.048), specifically phenylephrine (odds ratio: 1.7; 95% CI: 1.4–2.1; *P*<0.001). However, there were no differences in ephedrine use (odds ratio 0.9; 95% CI: 0.7–1.1; *P*=0.248). In other words, patients who received neoadjuvant chemotherapy received vasopressors and phenylephrine 20% and 70% more often than those who did not, respectively (Table 2).

**Table 2:** Differences in the use of vasopressors between groups. Data are presented as *n* (%) or median [first quartile–third quartile], as appropriate. ∗Odds ratio with 95% confidence interval (CI). ^†^Median of differences with 95% confidence interval using Hodges–Lehmann estimator. ^‡^Student's *t*-test or Wilcoxon rank-sum test, as appropriate.

Variable	No neoadjuvant chemotherapy	Neoadjuvant chemotherapy	Effect estimate (95% CI)	*P*-value^‡^
Use of vasopressors (*n*=1764)	491 (56%)	533 (60%)	1.22∗ (1–1.5)	0.048
Ephedrine (*n*=758)	391 (44%)	367 (42%)	0.9∗ (0.7–1.1)	0.248
	15 [10–25] mg	15 [10–25] mg	0^†^ (0–0) mg	0.177
Phenylephrine (*n*=786)	249 (28%)	357 (40%)	1.7∗ (1.4–2.1)	<0.001
	300 [100–500] μg	300 [150–600] μg	50^†^ (0–100) μg	0.072

**Fig 2:**
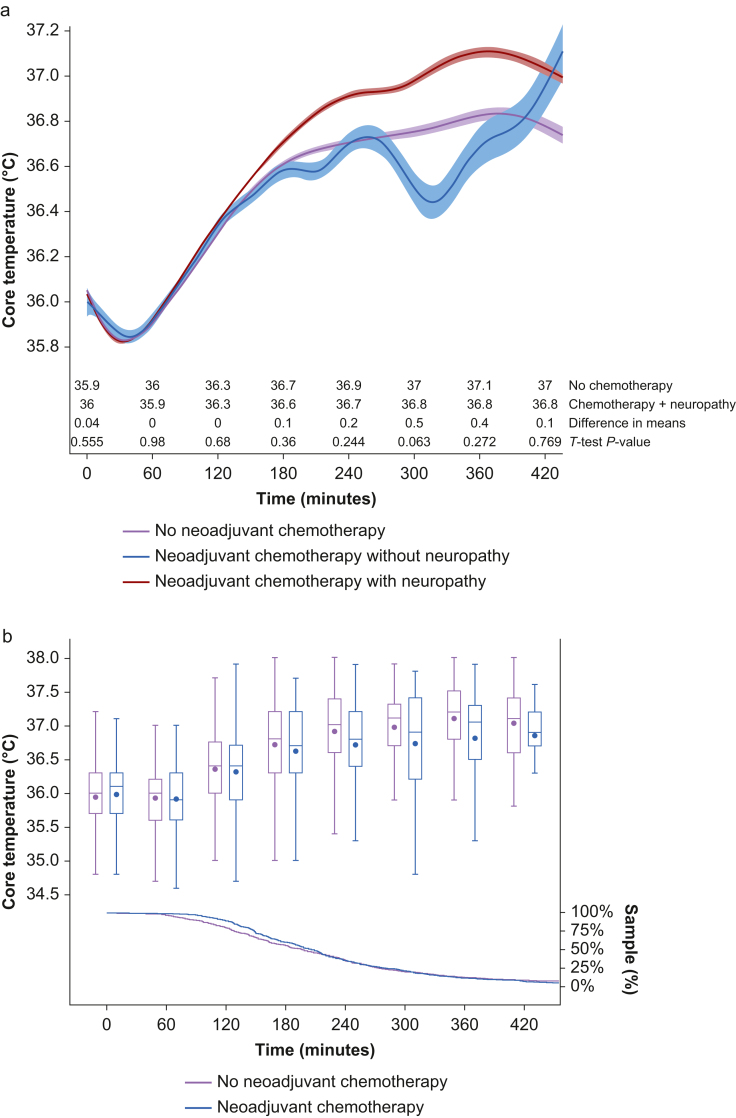
Comparison of core temperature between no neoadjuvant chemotherapy, neoadjuvant chemotherapy without neuropathy, and neoadjuvant chemotherapy with neuropathy groups using spline regression (represented by line) with 95% confidence interval (represented by band) as a function of time and hourly summary statistics (represented by box plot) with the proportion of sample data analysed over time (represented by line). (a) Comparison of core temperature over time between groups with hourly difference in means using Student's *t*-test. (b) Summary statistics (mean, median, inter-quartile interval, and minimum and maximum values) with proportion of sample data per group using Kaplan–Meier estimator.

Heart rate showed a similar distribution over time in both groups; however, the neoadjuvant chemotherapy group had an overall higher rate (Fig. 2a). The Wilcoxon–Mann–Whitney test suggests that the distribution of heart rate was different between the groups (*Z*=120.73; *P*<0.001). The proportion of validated heart rate measurements was higher than 50% of the sample for each group throughout the first 7 h after induction of anaesthesia (Fig. 2b).

### Subgroup analysis

Based on the finding that the incidence of neuropathy was three times higher in the neoadjuvant chemotherapy group, we conducted a *post hoc* analysis comparing patients with and without neuropathy within the neoadjuvant chemotherapy group (Supplementary Table 1). The results of this *post hoc* analysis should be regarded as hypothesis-generating.

**Fig 3:**
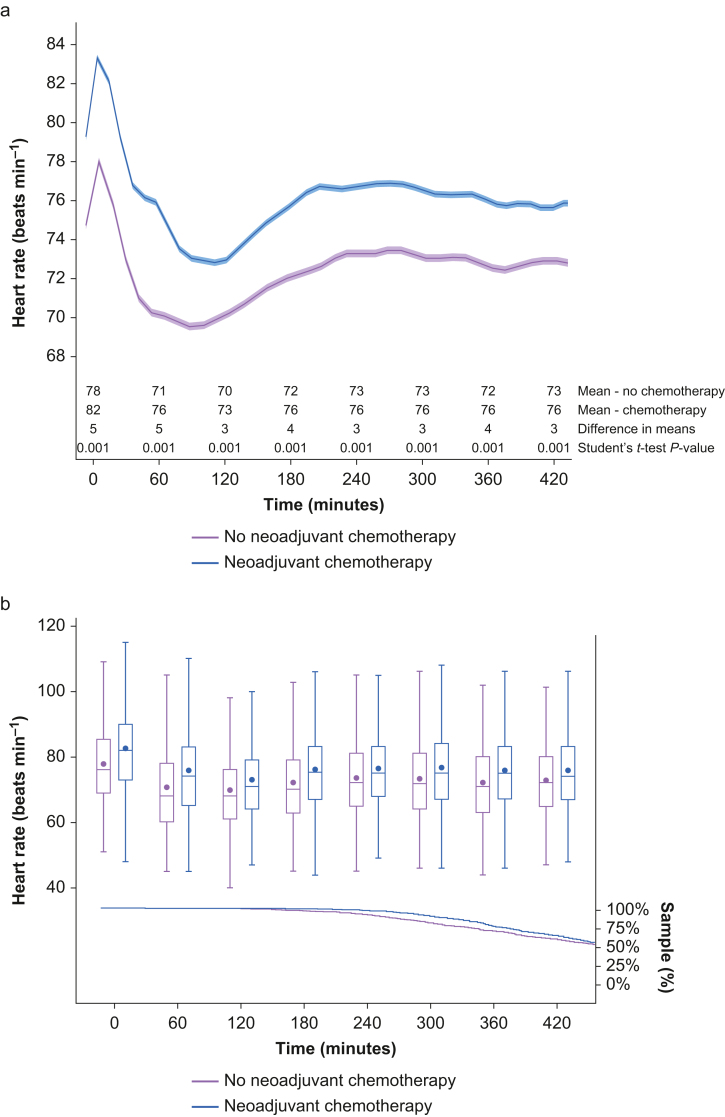
Comparison of intraoperative heart rate between no neoadjuvant chemotherapy *vs* neoadjuvant chemotherapy groups using spline regression (represented by line) with 95% confidence interval (represented by band) as a function of time and hourly summary statistics (represented by box plot) with the proportion of sample data analysed over time (represented by line). (a) Comparison of heart rate over time between groups with hourly difference in means using Student's *t*-test. (b) Summary statistics (mean, median, inter-quartile interval, and minimum and maximum values) with proportion of sample data per group using Kaplan–Meier estimator.

Patients with neuropathy presented with a similar pattern of intraoperative temperature within the first 2 h of anaesthesia. Interestingly, after the third hour, patients who received neoadjuvant chemotherapy and had neuropathy presented a distinct temperature distribution over time compared with patients without neuropathy and those who did not receive neoadjuvant chemotherapy, but the difference in means was not significant between no chemotherapy and neoadjuvant chemotherapy with neuropathy groups (Fig. 3a). Additionally, the proportion of validated temperature measurements fell below 25% of the sample for all groups close to 5 h after anaesthesia induction (Fig. 3b).

Vasopressors were administered during surgery in 80 (67%) patients with neuropathy and 479 (59%) patients without neuropathy in the neoadjuvant chemotherapy group. When compared with the no-neoadjuvant chemotherapy group, there was a statistically significant association between neuropathy and the use of vasopressors during surgery (odds ratio 1.6; 95% CI: 1.1–2.4; *P*=0.018). Accordingly, there was a difference in the odds ratio for phenylephrine use (odds ratio 2; 95% CI: 1.3–3; *P*<0.001), but no difference in ephedrine use (odds ratio 0.9; 95% CI: 0.7–1.4; *P*=0.949). In other words, patients who received neoadjuvant chemotherapy and had neuropathy received vasopressors and phenylephrine more often than those who did not received chemotherapy (Table 3).

**Table 3:** Differences in use of vasopressors in patients with neoadjuvant chemotherapy. Data are presented as *n* (%) or median [first quartile–third quartile], as appropriate. ∗Odds ratio with 95% confidence interval (CI). ^†^Median of differences with 95% CI using Hodges–Lehmann estimator. ^‡^χ^2^ independence test or Wilcoxon rank-sum test, as appropriate.

Variable	No neoadjuvant chemotherapy	Neoadjuvant chemotherapy without neuropathy	Neoadjuvant chemotherapy with neuropathy	Effect estimate (95% CI)	*P*-value^‡^
Use of vasopressors	—	479 (59%)	80 (67%)	1.4∗ (0.9–2.1)	0.089
	465 (56%)	—	80 (67%)	1.63∗ (1.1–2.4)	0.018
Ephedrine	—	331 (41%)	53 (44%)	1.16∗ (0.8–1.7)	0.441
		15 [10–25] mg	15 [10–25] mg	1.11^†^ (0.9–1.3) mg	0.302
	374 (45%)	—	53 (44%)	0.99∗ (0.7–1.4)	0.949
	15 [10–25] mg		15 [10–25] mg	1.15^†^ (0.9–1.4) mg	0.133
Phenylephrine	—	305 (40%)	52 (44%)	1.2∗ (0.8–1.7)	0.392
		300 [100–600] μg	400 [225–600] μg	75^†^ [0–150] μg	0.065
	233 (28%)	—	52 (44%)	2∗ (1.3–3)	<0.001
	300 [100–500] μg		400 [225–600] μg	100^†^ (0–200) μg	0.004

**Fig 4:**
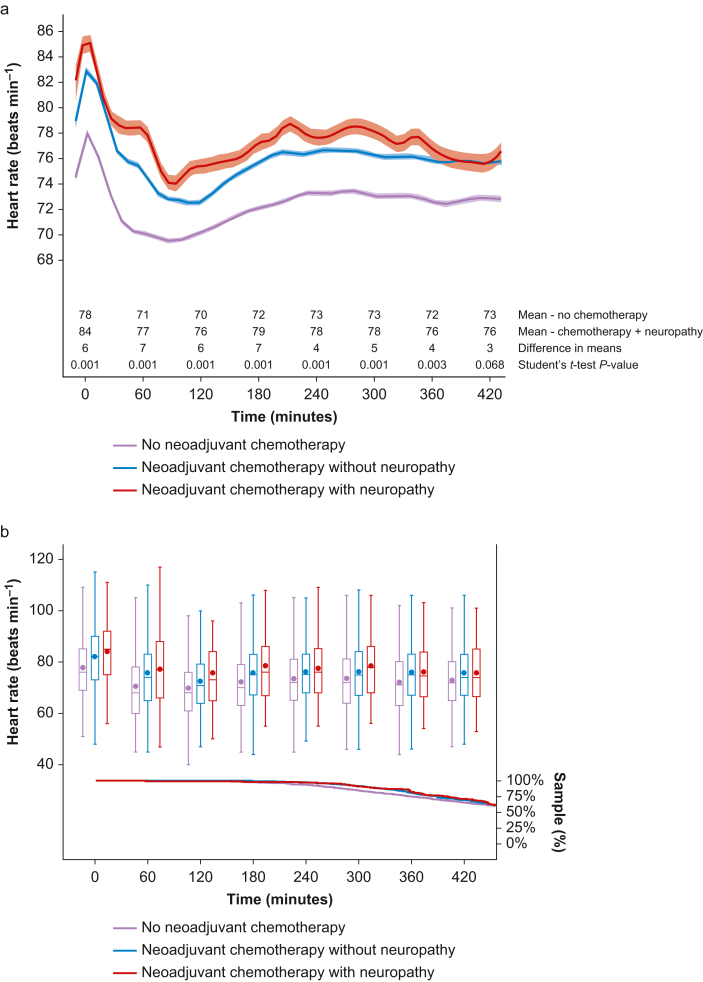
Comparison of intraoperative heart rate between no neoadjuvant chemotherapy, neoadjuvant chemotherapy without neuropathy, and neoadjuvant chemotherapy with neuropathy groups using spline regression (represented by line) with 95% confidence interval (represented by band) as a function of time and hourly summary statistics (represented by box plot) with the proportion of sample data analysed over time (represented by line). (a) Comparison of intraoperative heart rate over time between groups with hourly difference in means using Student's *t*-test. (b) Summary statistics (mean, median, inter-quartile interval, and minimum and maximum values) with proportion of sample data per group using Kaplan–Meier estimator.

Heart rate presented a similar distribution over time in all groups; however, patients with neuropathy in the neoadjuvant chemotherapy group presented an overall higher rate (Fig. 4a). The Kruskal–Wallis test suggested that there was a difference in intraoperative heart rate distribution between the groups (χ^2^: 15 128.97; degrees of freedom 2; *P*<0.001). Additionally, the proportion of validated heart rate measurements was higher than 50% of the sample for all groups throughout the first 7 h after induction of anaesthesia (Fig. 4b).

## Discussion

Our study shows that the administration of neoadjuvant chemotherapy for breast cancer is associated with differences in intraoperative plateau temperature in patients undergoing mastectomy, especially in longer procedures. Polynomial regression analysis revealed a narrow confidence interval, suggesting that intraoperative temperatures were adequately represented for each group. The mechanism associated with the initial decrease in temperature observed in both groups has been extensively studied and is largely associated with the redistribution of body heat resulting from anaesthesia-associated vasodilation, even in pre-warmed patients.^16^, ^17^ After the redistribution phase, both groups presented a similar rewarming rate; however, the neoadjuvant chemotherapy group did not reach the same plateau temperature, regardless of the duration of anaesthesia. According to the second law of thermodynamics, because both groups were exposed to similar conditions causing heat loss (e.g. from thoracic exposure) and heat gain (e.g. from forced air being warmer across the lower extremities) and presented a similar body surface area (Supplementary Table 1), we would expect similar plateau temperatures, especially after the initial heat redistribution phase. Therefore, it is plausible that the observed differences in plateau temperature were associated with neoadjuvant chemotherapy rather than other factors.

Hypotension is a common haemodynamic event that occurs during anaesthesia. Several studies have demonstrated an association between hypotension and critical incidents, such as kidney injury, myocardial injury, and death.^18^, ^19^ To reduce the risk of long-term complications, a common mitigating strategy involves using vasopressors to maintain blood pressure within acceptable levels during surgery.^20^ Because most of the study population was composed of adult females, the incidence of cardiovascular conditions detected by the Charlson Comorbidity Index and the Elixhauser Comorbidity Index was low and evenly balanced between groups, reducing the possibility of selection bias (Supplementary Tables 3 and 4). Thus, our study suggests that patients who received neoadjuvant chemotherapy required phenylephrine as vasopressor more often than those who did not receive chemotherapy.

In normal subjects, cardiovascular adaptation to ordinary physiological challenges is possible, mainly because of heart rate variations. Heart rate is primarily regulated by the balance between the sympathetic and parasympathetic nervous systems; however, the relationships are not linear.^21^ Given its high variability, heart rate is typically assessed by measuring the RR interval in milliseconds in a time or frequency domain or through other nonlinear methods.^22^ Because the electronic medical record system only saves the instantaneous heart rate measured by the pulse oximeter or ECG at constant intervals, it was not possible to evaluate heart rate variability. Interestingly, our study found a small but constant difference in heart rate, despite similar intraoperative fluid management, estimated blood loss, and comorbidity indices between groups.

The non-specific cytotoxic effects of chemotherapeutic agents are associated with adverse events, such as chemotherapy-induced peripheral neuropathy. The usual clinical presentation includes symmetrical peripheral sensory neuropathy with paraesthesia, dysaesthesia, and neuropathic pain with a classical ‘stocking and glove’ distribution.^23^ A recent publication associated peripheral neuropathy with mitochondrial oedema in both myelinated axons and small C fibres caused by microtubule-stabilising agents (paclitaxel or docetaxel) commonly used for neoadjuvant chemotherapy in breast cancer.^24^ Similarly, an experimental study concluded that vascular mechanosensitive dorsal root ganglion neurones are involved in maintaining cardiovascular homeostasis and that their ablation causes hypotension, increased heart rate variability, and death.^25^ Accordingly, the subgroup analysis demonstrated that within the neoadjuvant chemotherapy group, patients with neuropathy presented a different intraoperative temperature plateau pattern, required vasopressors more often, and exhibited higher heart rates than those without neuropathy. Perhaps the association between heterogeneous plateau temperatures, increased requirement of vasopressors, and higher heart rates in patients who received neoadjuvant chemotherapy suggests that neuropathy is associated with perioperative critical events. Our institution is currently conducting research to clarify this possible association.

We believe that such conclusions have not been reported previously because of the limited indications for neoadjuvant chemotherapy. A large meta-analysis conducted by the Early Breast Cancer Trialists' Collaborative Group concluded that neoadjuvant chemotherapy increased the frequency of breast-conserving surgery. However, no differences in disease-free survival or overall survival rates were observed.^5^ One study estimated that less than 20% of patients with newly diagnosed breast cancer received neoadjuvant chemotherapy between 2003 and 2011.^26^ Although multiple clinical assessment tools for the diagnosis of chemotherapy-induced neuropathy were published, the incidence of dysautonomia in patients receiving neoadjuvant chemotherapy is unknown because of the complexity of some diagnostic tests associated with a lack of consensus on the best assessment.^27^, ^28^ Because our institution is dedicated to cancer prevention, treatment, and research, chemotherapy-induced neuropathy is actively investigated and documented.

Intraoperative temperature assessment studies typically have good internal validity. As our study only included patients undergoing a specific type of surgery for similar indications, heat loss attributable to exposure and heat gain promoted by the forced-air warming system were similar in all patients. Because most patients were not hypothermic by the end of the surgery, there was a negligible incidence of complications associated with hypothermia, such as increased blood transfusion requirements and prolonged length of hospital stay. Late postoperative complications associated with hypothermia, such as wound infections and cardiovascular events, were not evaluated in the present study because they were not reliably documented in our database. Also, because the study population is largely composed of adult females with limited pre-surgical comorbidities, clinically relevant perioperative complications are less likely.

This study has some limitations, including the fact that our analysis was restricted to a single centre. As our institution is dedicated to cancer treatment, our patient population is more likely to present with severe and uncommon types of breast cancer, limiting the generalisability of these results. For example, the proportion of patients who received neoadjuvant chemotherapy in our study was almost twice the national average for the USA.^26^ Also, the proportion of validated core temperature data for each group decreased over time because the procedures we used to reduce data artifacts had a censoring effect: data generated with less than 25% of the sample should be considered with caution. Additionally, because the design of our study was retrospective, our results reflect associations and should not be interpreted as evidence of causality.

Although experimental studies have already demonstrated the pathophysiological mechanism of autonomic nervous system injury induced by chemotherapeutic agents,^29^ we could not find any previous studies that have investigated the effects of neoadjuvant chemotherapy on intraoperative measures during oncologic surgery, possibly because of the small proportion of patients receiving neoadjuvant chemotherapy for the treatment of breast cancer. In conclusion, our results provide additional evidence of the impact of neoadjuvant chemotherapy on autonomic function during general anaesthesia, especially in patients with neuropathy.

## Authors' contributions

Conceptualisation: DDK, JPC

Project administration: JPC

Supervision: JPC

Methodology: DDK, JPC

Investigation: DDK, JPC

Data curation: DDK

Formal analysis: PMD, JPC

Writing of original draft: DDK

Writing/review/editing: SMD, PMD, JPC

## Declarations of competing interest

The authors declare that they have no conflicts of interest.
