# Supplementary material for: Effect of neoadjuvant chemotherapy on intraoperative core temperature in patients with breast cancer: a retrospective cohort study
Source: BJA Open. 2022 Dec 31;5:100119. doi: 10.1016/j.bjao.2022.100119 (PMC10430839; doi:10.1016/j.bjao.2022.100119)
Supplement: Multimedia component 1 [file mmc1.docx]

Table 1. Preoperative hemoglobin levels, body surface area, and intraoperative fluid management

| **Variable** | **Before propensity matching** | | | | **After propensity matching** | | | |
| --- | --- | --- | --- | --- | --- | --- | --- | --- |
|  | **No chemotherapy** | **Chemotherapy** | **Effect estimate** | **p value ^d^** | **No chemotherapy** | **Chemotherapy** | **Effect estimate** | **p value^e^** |
| Preoperative hemoglobin (g/dl) | 13 ± 1.3  (n = 2,123) | 11.4 ± 1.3  (n = 852) | 1.6^b^  (1.5, 1.7) | < 0.001 | 12.9 ± 1.3  (n = 742) | 11.4 ± 1.3  (n = 852) | 1.5^b^  (1.4, 1.6) | < 0.001 |
| Body Surface Area ^a^ (m^2^) | 1.85 ± 0.2  (n = 2,527) | 1.84 ± 0.2  (n = 882) | 0.01^b^  (-0.01, 0.03) | 0.312 | 1.84 ± 0.2  (n = 882) | 1.84 ± 0.2  (n = 882) | 0.01^b^  (-0.1, 0.1) | 0.72 |
| Peripheral neuropathy | 123 (5%)  (n = 2,527) | 119 (13.5%)  (n = 882) | 3^c^  (2.3, 4) | < 0.001 | 48 (5.4%)  (n = 882) | 119 (13.5%)  (n = 882) | 2.7^c^  (1.9, 3.8) | < 0.001 |
| Crystalloids (ml) | 1,000 [800, 1500]  (n = 2,357) | 1,000 [900, 1600]  (n = 823) | 75^d^  (50, 100) | < 0.001 | 1,200 [825, 1700]  (n = 827) | 1,150 [900, 1600]  (n = 823) | 25^d^  (0, 50) | 0.605 |
| Colloids (ml) | 500 [500, 1000]  (n = 320) | 500 [500, 500]  (n = 145) | - | 0.085 | 500 [500, 1000]  (n = 143) | 500 [500, 500]  (n = 145) | - | 0.021 |
| Estimated blood loss (ml) | 40 [20, 75]  (n = 2,090) | 50 [25, 100]  (n = 778) | 5^d^  (0, 10) | < 0.001 | 50 [25, 100]  (n = 764) | 50 [25, 100]  (n = 778) | - | 0.384 |
| Packed red blood cell (ml) | 500 [394, 870]  (n = 13) | 457 [300, 814]  (n = 7) | 157^d^  (-107, 422) | 0.28 | 500 [417, 826]  (n = 9) | 457 [300, 814]  (n = 7) | 126^d^  (-117, 370) | 0.354 |

Data are presented as the mean ± standard deviation or median [1^st^ quartile, 3^rd^ quartile], as appropriate.

^a^ Mosteller method where body surface area in m^2^ = $\sqrt{\frac{(height \left( \mathrm{cm} \right). weight \left( \mathrm{kg} \right)}{3600}}$

^b^ Difference in means with 95% confidence interval

^c^ Odds ratio with 95% confidence interval

^d^ Median of differences with 95% confidence interval using Hodges Lehmann's estimator

^e^ Student’s T test, Chi-square independence test or Wilcoxon rank-sum test, as appropriate

Table 2. Surgical characteristics

| **Variable** | **Before propensity matching** | | | | **After propensity matching** | | | |
| --- | --- | --- | --- | --- | --- | --- | --- | --- |
|  | **No chemotherapy**  **(n = 2,524)** | **Chemotherapy**  **(n = 880)** | **Effect estimate^a^** | **p value^b^** | **No chemotherapy**  **(n = 882)** | **Chemotherapy**  **(n = 880)** | **Effect estimate^a^** | **p value^b^** |
| Radical mastectomy | 1,139  (45%) | 517  (59%) | 1.7  (1.5, 2) | < 0.001 | 475  (54%) | 517  (59%) | 1.2  (1, 1.5) | 0.039 |
| Simple mastectomy | 1,379  (55%) | 362  (41%) | 0.6  (0.5, 0.7) | < 0.001 | 405  (46%) | 362  (41%) | 0.82  (0.7, 1) | 0.044 |
| Bilateral mastectomy | 163  (55%) | 45  (41%) | 0.8  (0.6, 1.1) | 0.165 | 73  (8%) | 45  (5%) | 0.6  (0.4, 0.9) | 0.009 |
| Reconstruction | 1,289  (51%) | 430  (49%) | 0.9  (0.8, 1.1) | 0.273 | 529  (60%) | 430  (49%) | 0.6  (0.5, 0.8) | < 0.001 |
| Reconstruction with flap | 233  (9.2%) | 91  (10.3%) | 1.1  (0.9, 1.5) | 0.35 | 112  (13%) | 91  (10%) | 0.8  (0.6, 1.1) | 0.135 |
| Lymphadenectomy | 146  (5.8%) | 256  (29%) | 6.7  (5.3, 8.3) | < 0.001 | 67  (8%) | 256  (29%) | 5  (3.7, 6.6) | < 0.001 |

Data are presented as n (%)

^a^ Odds ratio with 95% confidence interval

^b^ Chi-square independence test

| **Variable** | **Before propensity matching** | | | | **After propensity matching** | | | |
| --- | --- | --- | --- | --- | --- | --- | --- | --- |
|  | **No chemotherapy**  **(n = 2,524)** | **Chemotherapy**  **(n = 880)** | **Effect estimate** | **p value^c^** | **No chemotherapy**  **(n = 882)** | **Chemotherapy**  **(n = 880)** | **Effect estimate** | **p value^c^** |
| Myocardial infarction | 31 (1.2%) | 11 (1.25%) | 1^a^  (0.5, 2) | 0.14 | 7 (0.8%) | 11 (1.2%) | 1.5^a^  (0.6, 4) | 0.478 |
| Congestive heart failure | 42 (1.7%) | 17 (1.9%) | 1.1^a^  (0.6, 2) | 0.654 | 13 (1.5%) | 17 (2%) | 1.3^a^  (0.6, 2.7) | 0.582 |
| Peripheral vascular disease | 35 (1.4%) | 8 (0.9%) | 0.6^a^  (0.3, 1.4) | 0.298 | 8 (1%) | 8 (1%) | 1^a^  (0.4, 2.6) | 1 |
| Cerebrovascular disease | 53 (2.1%) | 14 (1.6%) | 0.7^a^  (0.4, 1.3) | 0.399 | 13 (1.5%) | 14 (1.6%) | 1.1^a^  (0.5, 2.3) | 1 |
| Charlson Comorbidity score | 2 [0, 3] | 3 [2, 6] | 1.5^b^  (1, 2) | < 0.001 | 2 [0, 3] | 3 [2, 6] | 1.5^b^  (1, 2) | < 0.001 |

Table 3. Charlson Comorbidity Index

Data are presented as n (%) or median [1^st^ quartile, 3^rd^ quartile], as appropriate.

^a^ Odds ratio with 95% confidence interval

^b^ Median of differences with 95% confidence interval using Hodges Lehmann's estimator

^c^ Chi-square independence test or Wilcoxon rank-sum test, as appropriate

Table 4. Elixhauser Comorbidity Index

| **Variable** | **Before propensity matching** | | | | **After propensity matching** | | | |
| --- | --- | --- | --- | --- | --- | --- | --- | --- |
|  | **No chemotherapy**  (n = 2,496) | **Chemotherapy**  (n = 881) | **Effect estimate** | **p value^c^** | **No chemotherapy**  (n = 871) | **Chemotherapy**  (n = 880) | **Effect estimate** | **p value^c^** |
| Congestive heart failure | 42 (1.7%) | 17 (1.9%) | 1.1^a^  (0.6, 2) | 0.654 | 13 (1.5%) | 17 (1.9%) | 1.3^a^  (0.6, 2.7) | 0.582 |
| Cardiac arrhythmias | 246 (10%) | 70 (8%) | 0.8^a^  (0.6, 1) | 0.106 | 67 (8%) | 70 (8%) | 1^a^  (0.7, 1.5) | 0.859 |
| Valvular disease | 78 (3%) | 28 (3.2%) | 1^a^  (0.7, 1.6) | 0.911 | 20 (2.3%) | 28 (3.2%) | 1.4^a^  (0.8, 2.5) | 0.306 |
| Pulmonary circulation disorders | 35 (1.4%) | 15 (1.7%) | 1.2^a^  (0.7, 2.2) | 0.518 | 10 (1.1%) | 15 (1.7%) | 1.5^a^  (0.7, 3.3) | 0.421 |
| Peripheral vascular disorders | 35 (1.4%) | 8 (0.9%) | 0.6^a^  (0.3, 1.4) | 0.299 | 8 (1%) | 8 (1%) | 1^a^  (0.4, 2.6) | 1 |
| Hypertension, uncomplicated | 993 (40%) | 321 (36%) | 0.9^a^  (0.7, 1) | 0.084 | 261 (30%) | 321 (36%) | 1.3^a^  (1.1, 1.6) | 0.004 |
| Hypertension, complicated | 6 (0.2%) | 0 | 1^a^  (1, 1) | 0.349 | 1 (0.1%) | 0 | 1^a^  (1, 1) | 0.497 |
| Fluid and electrolyte disorders | 4 (0.2%) | 7 (0.8%) | 5^a^  (1.5, 17) | 0.01 | 0 | 7 (0.8%) | 1^a^  (1,1) | 0.015 |
| Elixhauser Comorbidity score | 6 [0, 7] | 7 [6, 13] | 4.5^b^  (4, 5) | < 0.001 | 6 [0, 7] | 7 [6, 13] | 4.5^b^  (4, 5) | < 0.001 |

Data are presented as the mean ± standard deviation or n (%), as appropriate.

^a^ Odds ratio with 95% confidence interval

^b^ Median of differences with 95% confidence interval using Hodges Lehmann's estimator

^c^ Chi-square independence test, Fisher’s exact test or Wilcoxon rank-sum test, as appropriate
