# Supplementary material for: Effect of neoadjuvant chemotherapy on intraoperative core temperature in patients with breast cancer: a retrospective cohort study
Source: BJA Open. 2022 Dec 31;5:100119. doi: 10.1016/j.bjao.2022.100119 (PMC10430839; doi:10.1016/j.bjao.2022.100119)
Supplement: Multimedia component 2 [file mmc2.docx]

STROBE Statement—checklist of items that should be included in reports of observational studies

|  | Item No. | Recommendation | Page  No. | Relevant text from manuscript |
| --- | --- | --- | --- | --- |
| **Title and abstract** | 1 | (*a*) Indicate the study’s design with a commonly used term in the title or the abstract | *1* | *“…Retrospective Cohort Study”* |
|  |  | (*b*) Provide in the abstract an informative and balanced summary of what was done and what was found | *3* | *“Methods… Results… Conclusions”* |
| Introduction | | | |  |
| Background/rationale | 2 | Explain the scientific background and rationale for the investigation being reported | *4* | *“Although autonomic disturbances have been well characterised in patients receiving adjuvant chemotherapy, little is known about the intraoperative consequences of neoadjuvant chemotherapy”* |
| Objectives | 3 | State specific objectives, including any prespecified hypotheses | *4-5* | *“We thus tested the primary hypothesis that neoadjuvant chemotherapy is associated with a lower core temperature. Secondarily, we tested the hypothesis that neoadjuvant chemotherapy is associated with increased vasopressor use and heart rate during anaesthesia for mastectomy.”* |
| Methods | | | |  |
| Study design | 4 | Present key elements of study design early in the paper | *6* | *“…retrospective cohort study of adult patients…* *study was approved by… Institutional Review Board”* |
| Setting | 5 | Describe the setting, locations, and relevant dates, including periods of recruitment, exposure, follow-up, and data collection | *6* | *“…patients who underwent mastectomy for breast cancer at our institution between April 2016 and July 2020… retrospective database study”* |
| Participants | 6 | (*a*) *Cohort study*—Give the eligibility criteria, and the sources and methods of selection of participants. Describe methods of follow-up  *Case-control study*—Give the eligibility criteria, and the sources and methods of case ascertainment and control selection. Give the rationale for the choice of cases and controls  *Cross-sectional study*—Give the eligibility criteria, and the sources and methods of selection of participants | *6* | *“…patients who underwent mastectomy for breast cancer at our institution between April 2016 and July 2020… We included patients with core temperatures measured during surgery”* |
|  |  | (*b*) *Cohort study*—For matched studies, give matching criteria and number of exposed and unexposed  *Case-control study*—For matched studies, give matching criteria and the number of controls per case | 7 | *“Propensity score matching was conducted to ensure that any potential presurgical confounding variables were balanced between the groups.”* |
| Variables | 7 | Clearly define all outcomes, exposures, predictors, potential confounders, and effect modifiers. Give diagnostic criteria, if applicable | 7-8 | *“The intraoperative temperature was measured at the bladder, nasopharynx, or distal oesophagus using standard clinical thermometers… Secondary outcomes included the use of any vasopressor (ephedrine or phenylephrine) during anaesthesia and the instantaneous heart rate measured by pulse oximetry or electrocardiography… neuropathy diagnosis was based on the International Statistical Classification of Diseases and Related Health Problems (ICD) codes registered in the patient´s medical records.”* |
| Data sources/ measurement | 8* | For each variable of interest, give sources of data and details of methods of assessment (measurement). Describe comparability of assessment methods if there is more than one group | *6-7* | *“The intraoperative temperature was measured at the bladder, nasopharynx, or distal oesophagus using standard clinical thermometers. The temperature values were automatically captured every minute using an electronic medical record system… Secondary outcomes included the use of any vasopressor… and the instantaneous heart rate measured by pulse oximetry or electrocardiography recorded every minute in the electronic medical record system… neuropathy diagnosis was based on the International Statistical Classification of Diseases and Related Health Problems (ICD) codes registered in the patient´s medical records.”* |
| Bias | 9 | Describe any efforts to address potential sources of bias | *6-8* | *“Patients diagnosed with diabetes mellitus before surgery were excluded because the disease can also cause dysautonomia and is independently associated with hypothermia during surgery… Propensity score matching was conducted to ensure that any potential presurgical confounding variables were balanced between the groups… To reduce artefactual data from temperature measurements, we followed previously published procedures”* |
| Study size | 10 | Explain how the study size was arrived at | 9 | *“A previous study reported a difference of 0.1°C between mean core temperatures of a large group of adult patients having non-cardiac surgery using forced warming and a subset of patients undergoing breast procedures, with a standard deviation of 0.6°C.”* |

Continued on next page

| Quantitative variables | 11 | Explain how quantitative variables were handled in the analyses. If applicable, describe which groupings were chosen and why | 8-9 | *“…intraoperative temperature was summarised using spline regression… the distribution was compared using Student´s t-test or the Wilcoxon-Mann-Whitney test, depending on data distribution… The use of vasopressors between groups was analysed as a categorical and as a continuous variable. For categorical analysis, the strength of association between groups was measured using Chi square test and the difference between groups was evaluated using Wilcoxon-Mann-Whitney test.”* |  |
| --- | --- | --- | --- | --- | --- |
| Statistical methods | 12 | (*a*) Describe all statistical methods, including those used to control for confounding | *7-9* | *“Propensity score matching was conducted to ensure that any potential presurgical confounding variables were balanced between the groups…Groups were compared using Student´s t-test or the Wilcoxon-Mann-Whitney test, depending on data distribution. All test were two-tailed and the cut-off level for statistical significance was 0.05 for both the primary and secondary outcomes”* |  |
|  |  | (*b*) Describe any methods used to examine subgroups and interactions | *8* | *“Additionally, we compared other relevant perioperative parameters not included in the propensity matching to reduce the chances of overfitting the logistic regression model.”* |  |
|  |  | (*c*) Explain how missing data were addressed | *6* | *“Patients with duplicated or incomplete data… in the electronic medical record system were excluded”* |  |
|  |  | (*d*) *Cohort study*—If applicable, explain how loss to follow-up was addressed  *Case-control study*—If applicable, explain how matching of cases and controls was addressed  *Cross-sectional study*—If applicable, describe analytical methods taking account of sampling strategy | *N/A* | *N/A* |  |
|  |  | (*e*) Describe any sensitivity analyses | *N/A* | *N/A* |  |
| Results | | | | | |
| Participants | 13* | (a) Report numbers of individuals at each stage of study—eg numbers potentially eligible, examined for eligibility, confirmed eligible, included in the study, completing follow-up, and analysed | *10* | *“In total, 3,409 patients were included in the study (2,527 in the no-chemotherapy group and 882 in the neoadjuvant chemotherapy group)… After propensity score matching, 1,764 patients were successfully matched (882 in each group).”* |  |
|  |  | (b) Give reasons for non-participation at each stage | *N/A* | *N/A* |  |
|  |  | (c) Consider use of a flow diagram | *N/A* | *N/A* |  |
| Descriptive data | 14* | (a) Give characteristics of study participants (eg demographic, clinical, social) and information on exposures and potential confounders | *10* | *Supplement tables 1, 2, 3, and 4* |  |
|  |  | (b) Indicate number of participants with missing data for each variable of interest | *11-12* | *Tables 2 and 3* |  |
|  |  | (c) *Cohort study*—Summarise follow-up time (eg, average and total amount) | *N/A* | *N/A* |  |
| Outcome data | 15* | *Cohort study*—Report numbers of outcome events or summary measures over time | *11-12* | *Tables 2 and 3* |  |
|  |  | *Case-control study—*Report numbers in each exposure category, or summary measures of exposure |  |  |  |
|  |  | *Cross-sectional study—*Report numbers of outcome events or summary measures |  |  |  |
| Main results | 16 | (*a*) Give unadjusted estimates and, if applicable, confounder-adjusted estimates and their precision (eg, 95% confidence interval). Make clear which confounders were adjusted for and why they were included | *11-12* | *Tables 2 and 3, Supplement table 1* |  |
|  |  | (*b*) Report category boundaries when continuous variables were categorized | *11-12* | *Tables 2 and 3, Supplement table 1* |  |
|  |  | (*c*) If relevant, consider translating estimates of relative risk into absolute risk for a meaningful time period | N/A | N/A |  |

Continued on next page

| Other analyses | 17 | Report other analyses done—eg analyses of subgroups and interactions, and sensitivity analyses | *11* | *“Subgroup analysis”* | |
| --- | --- | --- | --- | --- | --- |
| Discussion | | | | |  |
| Key results | 18 | Summarise key results with reference to study objectives | *13-14* | *“Our study shows that the administration of neoadjuvant chemotherapy for breast cancer is associated with differences in intraoperative plateau temperature in patients undergoing mastectomy, especially in longer procedures… our study suggests that patients who received neoadjuvant chemotherapy required phenylephrine as vasopressor more often than those who did not receive chemotherapy… Interestingly, our study found a small but constant difference in heart rate, despite similar intraoperative fluid management, estimated blood loss, and comorbidity indexes between groups”* | |
| Limitations | 19 | Discuss limitations of the study, taking into account sources of potential bias or imprecision. Discuss both direction and magnitude of any potential bias | *16* | *“Late postoperative complications associated with hypothermia… were not evaluated in the present study because they were not reliably documented in our database. This study has some limitations, including the fact that our analysis was restricted to a single center… the proportion of validated core temperature data for each group decreased over time due to censoring effect of the artefactual data reducing procedure”* | |
| Interpretation | 20 | Give a cautious overall interpretation of results considering objectives, limitations, multiplicity of analyses, results from similar studies, and other relevant evidence | *15-16* | *“We believe that such conclusions have not been reported previously because of the limited indications for neoadjuvant chemotherapy… because the design of our study was retrospective, our results reflect associations and should not be interpreted as evidence of causality. Although experimental studies have already demonstrated…, we could not find any previous studies that have investigated the effects of neoadjuvant chemotherapy…, possibly because of the small proportion of patients receiving neoadjuvant chemotherapy for the treatment of breast cancer”* | |
| Generalisability | 21 | Discuss the generalisability (external validity) of the study results | *16* | *“since the study population is largely composed of adult females with limited presurgical comorbidities, clinically relevant perioperative complications are less likely… As our institution is dedicated to cancer treatment, our patient population is more likely to present with severe and uncommon types of breast cancer limiting the generalisability of results.”* | |
| Other information | |  | | |  |
| Funding | 22 | Give the source of funding and the role of the funders for the present study and, if applicable, for the original study on which the present article is based | *17* | *“This research did not receive any specific grants from funding agencies in the public, commercial, or not-for-profit sectors”* | |

*Give information separately for cases and controls in case-control studies and, if applicable, for exposed and unexposed groups in cohort and cross-sectional studies.

**Note:** An Explanation and Elaboration article discusses each checklist item and gives methodological background and published examples of transparent reporting. The STROBE checklist is best used in conjunction with this article (freely available on the Web sites of PLoS Medicine at http://www.plosmedicine.org/, Annals of Internal Medicine at http://www.annals.org/, and Epidemiology at http://www.epidem.com/). Information on the STROBE Initiative is available at www.strobe-statement.org.
